# Deep-learning-derived input function in dynamic [^18^F]FDG PET imaging of mice

**DOI:** 10.3389/fnume.2024.1372379

**Published:** 2024-04-11

**Authors:** Samuel Kuttner, Luigi T. Luppino, Laurence Convert, Otman Sarrhini, Roger Lecomte, Michael C. Kampffmeyer, Rune Sundset, Robert Jenssen

**Affiliations:** ^1^ The PET Imaging Center, University Hospital of North Norway, Tromsø, Norway; ^2^ UiT Machine Learning Group, Department of Physics and Technology, UiT The Arctic University of Norway, Tromsø, Norway; ^3^ Nuclear Medicine and Radiation Biology Research Group, Department of Clinical Medicine, UiT The Arctic University of Norway, Tromsø, Norway; ^4^ Sherbrooke Molecular Imaging Centre of CRCHUS and Department of Nuclear Medicine and Radiobiology, Université de Sherbrooke, Sherbrooke, QC, Canada; ^5^ Imaging Research & Technology Inc., Sherbrooke, QC, Canada

**Keywords:** dynamic positron emission tomography (PET), small-animal PET 18F-FDG PET/CT, Patlak analysis, arterial input function estimation, glucose metabolism, deep learning, prediction model

## Abstract

Dynamic positron emission tomography and kinetic modeling play a critical role in tracer development research using small animals. Kinetic modeling from dynamic PET imaging requires accurate knowledge of an input function, ideally determined through arterial blood sampling. Arterial cannulation in mice, however, requires complex, time-consuming and terminal surgery, meaning that longitudinal studies are impossible. The aim of the current work was to develop and evaluate a non-invasive, deep-learning-based prediction model (DLIF) that directly takes the PET data as input to predict a usable input function. We first trained and evaluated the DLIF model on 68 [^18^F]Fluorodeoxyglucose mouse scans with image-derived targets using cross validation. Subsequently, we evaluated the performance of a trained DLIF model on an external dataset consisting of 8 mouse scans where the input function was measured by continuous arterial blood sampling. The results showed that the predicted DLIF and image-derived targets were similar, and the net influx rate constants following from Patlak modeling using DLIF as input function were strongly correlated to the corresponding values obtained using the image-derived input function. There were somewhat larger discrepancies when evaluating the model on the external dataset, which could be attributed to systematic differences in the experimental setup between the two datasets. In conclusion, our non-invasive DLIF prediction method may be a viable alternative to arterial blood sampling in small animal [^18^F]FDG imaging. With further validation, DLIF could overcome the need for arterial cannulation and allow fully quantitative and longitudinal experiments in PET imaging studies of mice.

## 1 Introduction

Small animal positron emission tomography (PET) is a non-invasive medical imaging tool that is essential in the development of new molecular imaging tracers, drugs, diagnostic procedures and disease therapies (1–3) In particular, dynamic PET imaging plays a critical role in the imaging of small animals, as it can visualize the time-dependent tracer uptake *in vivo*, and by the application of tracer kinetic modeling, it allows quantification of biochemical processes, such as glucose metaolism (4). Specifically for irreversible tracers, Patlak analysis can be applied to compute the influx rate constant, $K_{i}$, which, for [^18^F]Fluorodeoxyglucose ([^18^F]FDG), is proportional to the metabolic rate of glucose (5, 6). The advantage of the Patlak model is that it may provide faster and more accurate calculations on voxel-wise, parametric images of $K_{i}$, compared to full tracer kinetic modeling (7, 8). A prerequisite for both tracer kinetic modeling and Patlak analysis is that both the tissue uptake curve and the time-activity curve of the tracer in the blood, known as the arterial inut function (AIF), are known (9).

In preclinical PET imaging of rodents, arterial blood sampling is considered the gold-standard method to sample the input function. However, it requires complex and time-consuming surgery to insert an arterial catheter and allows only a limited blood volume to be withdrawn, without altering the animal physiology (10). Longitudinal experiments with arterial blood sampling from the superficial branch of the femoral artery has been described in the literature for rats (11–13). For mice, however, the most common approach is to cannulate the carotid artery (14). This usually requires terminal surgery because the animals cannot be awakened after the experiments, meaning that longitudinal studies with arterial blood sampling in mice are impossible. To overcome these shortcomings, several alternative methods have been proposed, including the use of a population-based AIF template (15), image-derived input function (IDIF) (16), and simultaneous estimation (17, 18). Although these methods overcome the need for arterial blood sampling, they still have limited practical usability. For instance, the population-based approach neglects individual physiological differences and scan-dependent variations, and requires at least one blood sample for curve scaling. The IDIF approach must be corrected for the limited spatial and temporal resolution of the PET imaging system, image noise, and cardiac and respiratory motion (10, 19–21). Simultaneous estimation could estimate both the AIF and kinetic parameters, but it assumes a known mathematical AIF model and requires at least one late blood sample for parameter estimation (17, 18, 22, 23).

Machine learning and deep learning methods have been used increasingly in recent years for many medical applications, including segmentation, classification, and regression problems (24). Specifically, we have proposed the use of Gaussian Processes and long-short-term-memory (LSTM) models for AIF estimation (25, 26). These models required time-activity curves from up to five tissue regions as model input for the prediction of the AIF. Thus, our earlier approaches were limited by the need for manual delineation of several regions of interest as input, which implies domain specific knowledge, and most importantly, long data processing time. An alternative AIF estimation method is the multilayer 3D-residual network with a regression module, proposed in (27). This offers a promising similarity in shape to the IDIF, though it faced challenges predicting some parts of the curve. Recently, a combined deep-learning-based and model-based method was proposed to estimate the parameters of the input function (28), however this model was only evaluated in a phantom study, and it assumes a specific mathematical AIF model. Nevertheless, there is a need to overcome the limitations associated with the aforementioned methods for sampling or estimating the arterial input function, which would increase practical usability of quantitative PET imaging and allow for longitudinal studies of mice.

In this work, we propose a deep learning model designed to take the four-dimensional PET data as input and a deep-learning-derived input function (DLIF) as output. The approach avoids the need of tedious and time-consuming manual segmentation of regions of interest, has no assumptions of a specific AIF model, and does not require any blood samples for calibration purposes. We evaluate the model by comparing the predicted input function to the reference ones, as well as using both voxel-wise and regional Patlak analysis. Thus, by overcoming the drawbacks of blood sampling and the limitations of other AIF estimation techniques, with proper validation, DLIF would allow for fully quantitative and longitudinal PET imaging of mice, and as such provide an instrumental step forward for quantitative small animal imaging research.

## 2 Methods

### 2.1 Datasets

The small animal imaging data used to train and evaluate the DLIF model was collected in retrospect from two independent cohorts, acquired at UiT The Arctic University of Norway (UiT) and Université de Sherbrooke (UdS). The same PET imaging system and radiotracer was used at both centers, however, there were some systematic differences in the experimental methods and available animal strains in the datasets (Table 1). The UiT dataset was used for training and initial evaluation of the model. Because a ground truth AIF was unavailable in this dataset, an IDIF was used as training targets. On the other hand, in the UdS dataset continuous arterial blood sampling was performed simultaneously with PET acquisition. This dataset was used for additional evaluation of the DLIF model.

**Table 1:** Overview of the datasets in the UiT and UdS cohorts.

	UiT	UdS
Number of animals		
Balb/c	17	3
NZBWF1	51	–
C57/BL/6	–	2
CD-1	–	3
Total number of animals	68	8
Age [weeks]	24 ± 8	n/a
Weight [g]	33±8	31±3
Fasting time	3 h 50 min ±20 min	No fasting
Time in anaesthesia prior to PET	1 h 17 min ±19 min	45 min ±13 min
Blood glucose [mmol/L]	6.9±1.6	9.0±3.5
Injected dose [MBq]	10.5±1.8	10.0±3.3
Input function	IDIF	AIF

#### 2.1.1 UiT dataset

*Animals and preparations*

Preclinical PET/computed tomography (CT) data of 68 mouse scans were collected in retrospect from an already completed research study (29). This animal study was approved by the Competent Authority on Animal Research, the Norwegian Food Safety Authority; FOTS id 6676/2015. Thirty-six female mice from two strains [NZBWF1, Jax stock # 10008 (n=24)] and [BALB/cAnNCrl (n=12)], purchased from The Jackson Laboratory and Charles River Laboratories, respectively, were included. The mice were fasted for 3 h 50 min ±20 min, weighed, and anesthetized for 1 h 17 min ±19 min prior to tracer injection in an oxygen-isoflurane mixture (4% and 2% isoflurane for induction and maintenance, respectively), to reduce animal stress and allow stabilization of breathing and heart rate (29). Blood glucose was measured in venous blood to 6.9 mmol/L ±1.6 mmol/L prior to tracer administration, using a glucose meter (FreeStyle Lite, Abott Laboratories). A catheter, made from polyethylene tubing and a 30 gauge needle, was placed into the caudal vein to allow tracer injection.

*Image acquisition*

The PET/CT scans were performed using a Triumph^TM^ LabPET-8^TM^ small animal PET/CT scanner. Each mouse was scanned between 1–5 times at different ages (range 7–37 weeks), weighing 33±8 g at imaging time. The anesthetized mice were centered in the field-of-view of the PET/CT scanner, while lying on a 35 ^∘^C heated bed inside an animal imaging cell, with sensors monitoring heart and breathing rate. Tracer administration was conducted by the injection of 10.5±1.8 MBq [^18^F]FDG in 100 μl sterile saline through a tail-vein catheter during 30 s. For 56 scans, injections were performed with an infusion pump, while 12 scans were injected manually followed by 20 μl flush of sterile saline. A 60-min list-mode PET acquisition was started at injection time, followed by CT imaging for PET attenuation and scatter correction.

*Image reconstruction and processing*

The PET images were reconstructed into 44 time frames (24×5, 9×20, and 11×300 s) using a 3-dimensional maximum-likelihood estimator algorithm with 50 iterations. Corrections for detector efficiency, radioactive decay, random coincidences, dead time, attenuation and scatter were applied. The voxels were normalized into standardized uptake value (SUV) [g/ml] (30). Each time frame had an image matrix size of 128×92×92 voxels. In the current study, only time points up to 45 min were included to match the external UdS dataset, so the last three time frames from the reconstruction were discarded in all following analysis. Furthermore, in order to reduce the memory load during model training, the image dimensions were cropped to 64×48×48, which still encompassed the most vital regions of the mouse.

*Volume of interest delineation*

Volumes of interest were delineated using PMOD 3.8 (PMOD Technologies Ltd.) in either dynamic PET or static PET space, the latter which was formed by averaging the last 20 min of the dynamic PET acquisition. Delineations of vena cava, myocardium, left ventricle and brain were performed in a standardized and reproducible way, as described in (25). In short, vena cava was defined in a 0.6 mm radius sphere centered on a peak voxel in an early time step of the dynamic PET sequence; myocardium was delineated as voxels above 40–60% of the max voxel value above background in the whole heart in static PET space; left ventricle was defined as the region encompassed by the myocardium uptake; and brain was delineated as a 2 mm radius sphere in the dorsal region of the skull, visually identified in static PET space. All VOIs were applied to the dynamic PET images, and the mean time-activity curve was extracted from each VOI.

*IDIF calculation*

The IDIF targets for each mouse scan was formed from a parameterized model fit to image-derived data points derived from vena cava and left ventricle volume-of-interest, as described in (25).

#### 2.1.2 UdS dataset

*Animals and preparations*

The UdS dataset consisted of eight female mice from three different strains (C57/BL/6 (n=2), CD-1 (n=3), and BALB/c (n=3)). Four mice were purchased from Charles River Laboratories, while four were donations from the central animal facility with unknown origin. Animal experiments were performed following the recommendations of the Canadian Council on Animal Care and were approved by the Université de Sherbrooke in-house Ethics Committee for Animal Experiments under Protocol 2022–3463. Animals had free access to food and water before the experiments. Animals were anesthetized with isoflurane (2% +1.5 L/min O_2_) for 45 min ±13 min prior to tracer injection, while being cannulated in the caudal vein for tracer injection (prefilled with heparinized saline, 0.9%, 50 U/ml), and in the carotid artery for blood withdrawal, as described in (14). The animal temperature was regulated, and the heartbeat and breathing were monitored to ensure that physiological conditions were maintained as stable during the scan. Animal weight was 31±3 g at imaging time. Blood glucose was measured in venous blood to 9.0 mmol/L ±3.5 mmol/L prior to tracer administration.

*Image acquisition and arterial blood sampling*

The anesthetized mice were placed in a Triumph^TM^ LabPET-8^TM^ small animal PET/CT scanner with the heart centered in the field of view. An ultrahigh sensitivity blood counter (UHS-BC) was placed on a table in front of the scanner (detector to animal distance 60 cm). The mice were injected with 10.0±3.3 MBq of [^18^F]FDG in 100–200 μl sterile saline during 30 s through a 20 cm long PE10 catheter. The withdrawal pump speed was set to 15μl/min up to 5 min, then 7 μl/min for 36 min, corresponding to a total of 327 μl (15%) blood loss during the imaging experiment. This is within the maximum recommended blood loss from a single blood sample study (31). A 45-min list-mode PET acquisition was started at injection time. For the UdS data, CT imaging for PET attenuation and scatter correction was not performed.

*Image reconstruction and processing and analysis*

The PET images were reconstructed into 41 time frames, using the same framing, reconstruction algorithm and image corrections as described for the UiT dataset. However, because CT imaging was not available for the UdS data, attenuation and scatter correction was not performed. The measured blood curve was corrected for decay, delay, dead time and dispersion, as described in (14). Volumes of interest and an IDIF was also generated for this dataset, in a similar manner as for the UiT data.

### 2.2 Model architecture

**Figure 1:**
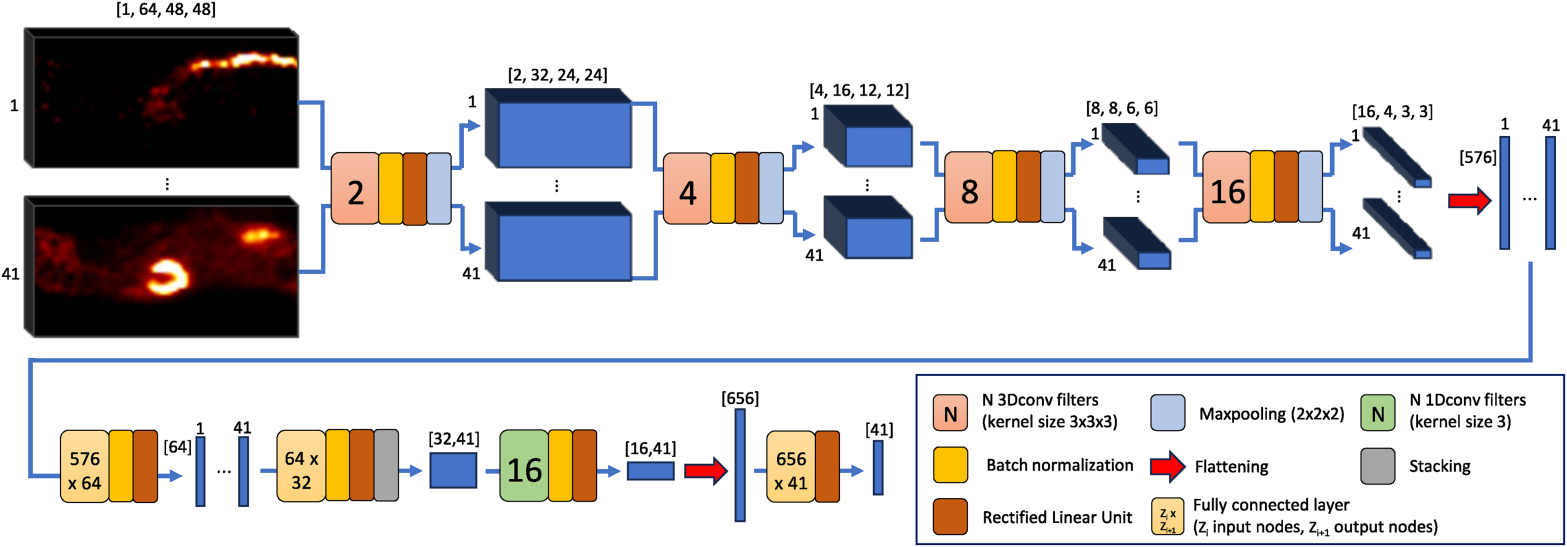
The architecture of the DLIF prediction model. First, four three-dimensional convolutional layers followed by two fully connected layers reduce the size of the volumes associated to each time frame. Then, one-dimensional convolutional filters capture the temporal correlations between neighboring time frames, and finally a fully connected layer outputs the DLIF. Single numbers indicate the corresponding time frames, while numbers in square brackets indicate the data size.

The proposed DLIF model architecture is shown in Figure 1. First, the spatial dimensions of each of the 41 volumes associated to each of the time frames of a single sample are reduced, while at the same time relevant spatial information is extracted and noise filtered out. The main expedient consists of feeding the volumes in parallel to four layers of 3D convolutional filters coupled with batch normalization, rectified linear unit (ReLU) activation functions and finally, 3D maxpooling layers. Choices of the different components are well described in the literature (32), but for instance, batch normalization has been shown to speed up the training process and act as a regularizer to avoid overfitting (33), while ReLU seems the most reasonable choice for activation function when the input and output data are non-negative quantities (34). Also, halving the spatial dimensions with maxpooling while doubling the number of filters for each layer is a well-known approach for obtaining a rich yet compact representation of the input, and many well-known architectures adopt this strategy (35, 36). Once the smallest spatial dimensions are reached (4×3×3), these 41 volumes of 16 features are flattened, so that each time frame is represented by a vector of 576 features. These vectors are reduced to a length of 32 by two cascaded multilayer perceptrons. Subsequently, 16 filters of 1D convolution are applied along the time axis, to capture the temporal correlations among the neighboring time frames. Finally, a multilayer perceptron takes the 16×41 extracted features and outputs a 41 long vector as output, representing the predicted DLIF.

### 2.3 Training regimes

Two different training regimes were used. First, the DLIF model was trained and evaluated with the UiT dataset, using 17-fold cross validation. Cross validation is commonly applied for model performance evaluation in settings with limited available training data (37). By iteratively splitting the dataset to use 64 samples for training and 4 samples for testing in each of the 17 folds, this allowed to have all 68 available samples in the test set once. Next, a new DLIF model was trained using all samples from the UiT dataset, and subsequently tested on the UdS dataset. In this setting, 50 runs with different random initializations were performed to obtain statistics over the DLIF predictions.

In both training regimes the Adam optimizer (38) with standard hyperparameters was selected to perform the minimization of a mean square error loss between the ground truth and the DLIF. Training was performed for 200 epochs with a learning rate of $2\cdot 10^{-4}$.

The DLIF models were implemented in Python 3.11.5, using PyTorch 2.1.0.

### 2.4 Model evaluation and statistical analyses

For both training regimes, the DLIF-predicted curves were first compared point by point to the respective reference input function using orthogonal regression. Orthogonal regression was chosen when comparing the measured and the predicted input function during regression analysis, because it assumes measurement error in both variable pairs, as opposed to standard linear regression, which assumes measurement error in only the independent variable (39). Patlak modeling (5, 6) was implemented in an in-house developed script (Python 3.11.5) and used to calculate the influx rate constant, $K_{i}$, for each voxel, as well as for brain and myocardium tissues, using both the predicted DLIF and each reference input function. For the UdS dataset, the regional influx rate constants for brain and myocardium tissues were also calculated using the IDIF and compared to those obtained with the AIF.

DLIF- and IDIF-based influx estimates were compared to those obtained from respective reference input function using paired t-test (α=0.05) and orthogonal regression. Normality was assessed using quantile-quantile plots. All statistical analyses were implemented in an in-house developed script (Python 3.11.5).

## 3 Results

Two datasets were used in these experiments. The UiT dataset, consisting of 68 mouse PET scans and an IDIF as reference input function, was used to train and evaluate the model through 17-fold cross validation. Next, the UiT dataset was used as a whole for training while the UdS dataset, consisting of 8 mouse PET scans with corresponding AIF from arterial blood sampling, was used as an external test set.

### 3.1 Cross-validation

**Figure 2:**
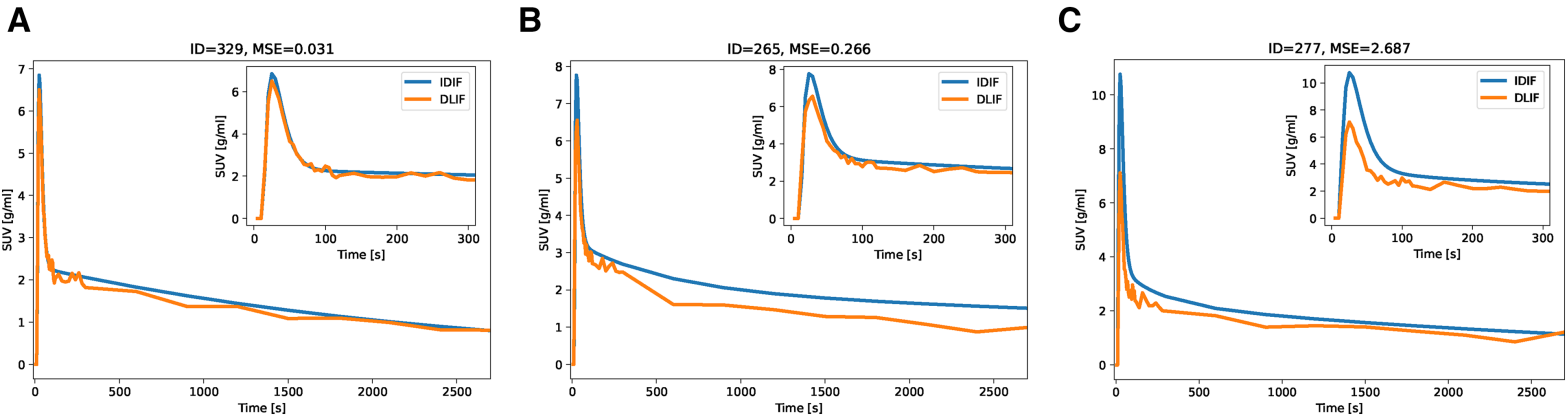
IDIF and predicted DLIF during cross validation experiments. The DLIF was predicted for each mouse scan when it was in the test set during each fold. (**A**) IDIF and DLIF for the mouse scan with the lowest error. (**B**) IDIF and DLIF for the mouse scan with the 50-percentile error. (**C**) IDIF and DLIF for the mouse scan with the largest error.

**Figure 3:**
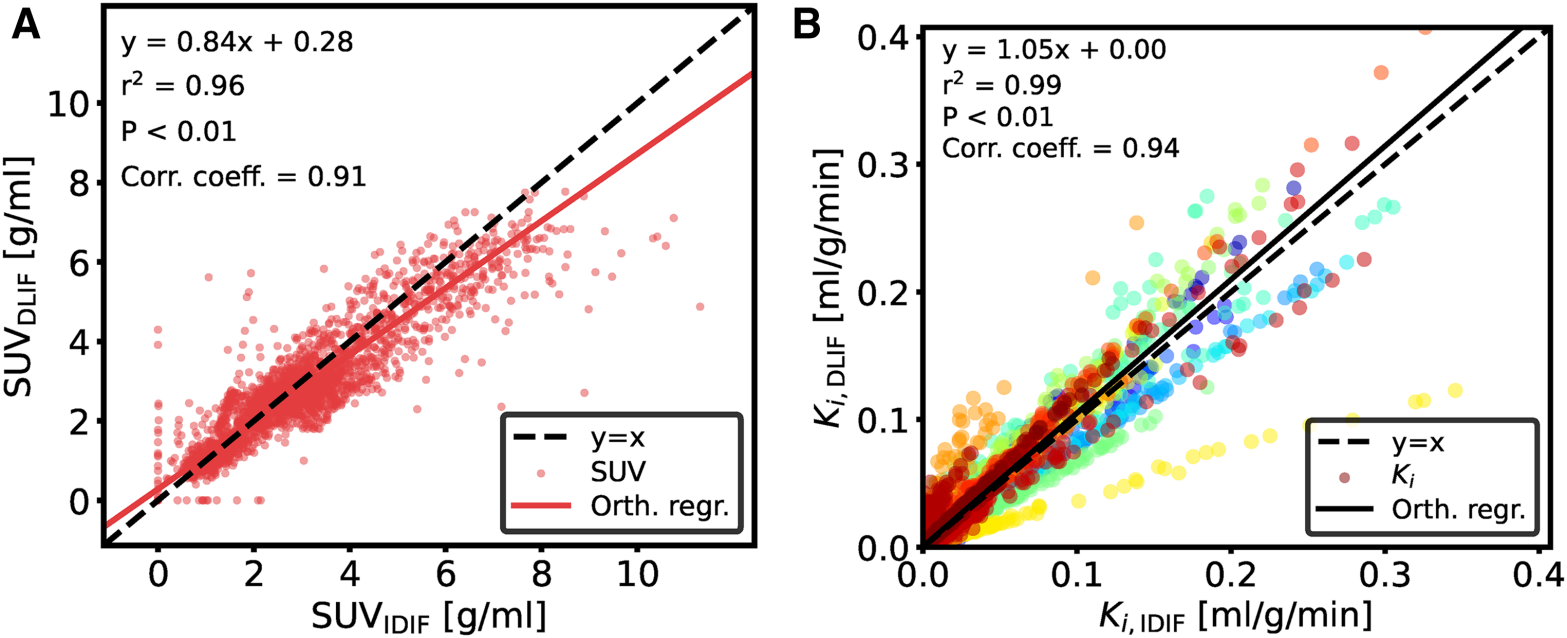
Comparison of data points between (**A**) DLIF and IDIF curves and (**B**) voxelwise Patlak using DLIF and IDIF as input function, respectively. The DLIF was predicted for each mouse scan when it was in the test set during each fold. Due to a large number of data points in (**B**), for visualization purposes, only 1,000 randomly sampled data points are shown. The color cycle indicates each mouse scan.

The overall input function curve shape, with an early peak and a vanishing tail, is captured by the DLIF model, as shown for the three mouse scan examples in Figure 2. Investigating the data points from all samples, there is a strong overall linear relationship (slope: 0.84) and strong correlation (correlation coefficient: 0.91) between DLIF and IDIF (Figure 3A). The DLIF model slightly underestimates the IDIF peak, shown as a deviation from the linear model for data points with high SUV value in Figure 3A.

**Figure 4:**
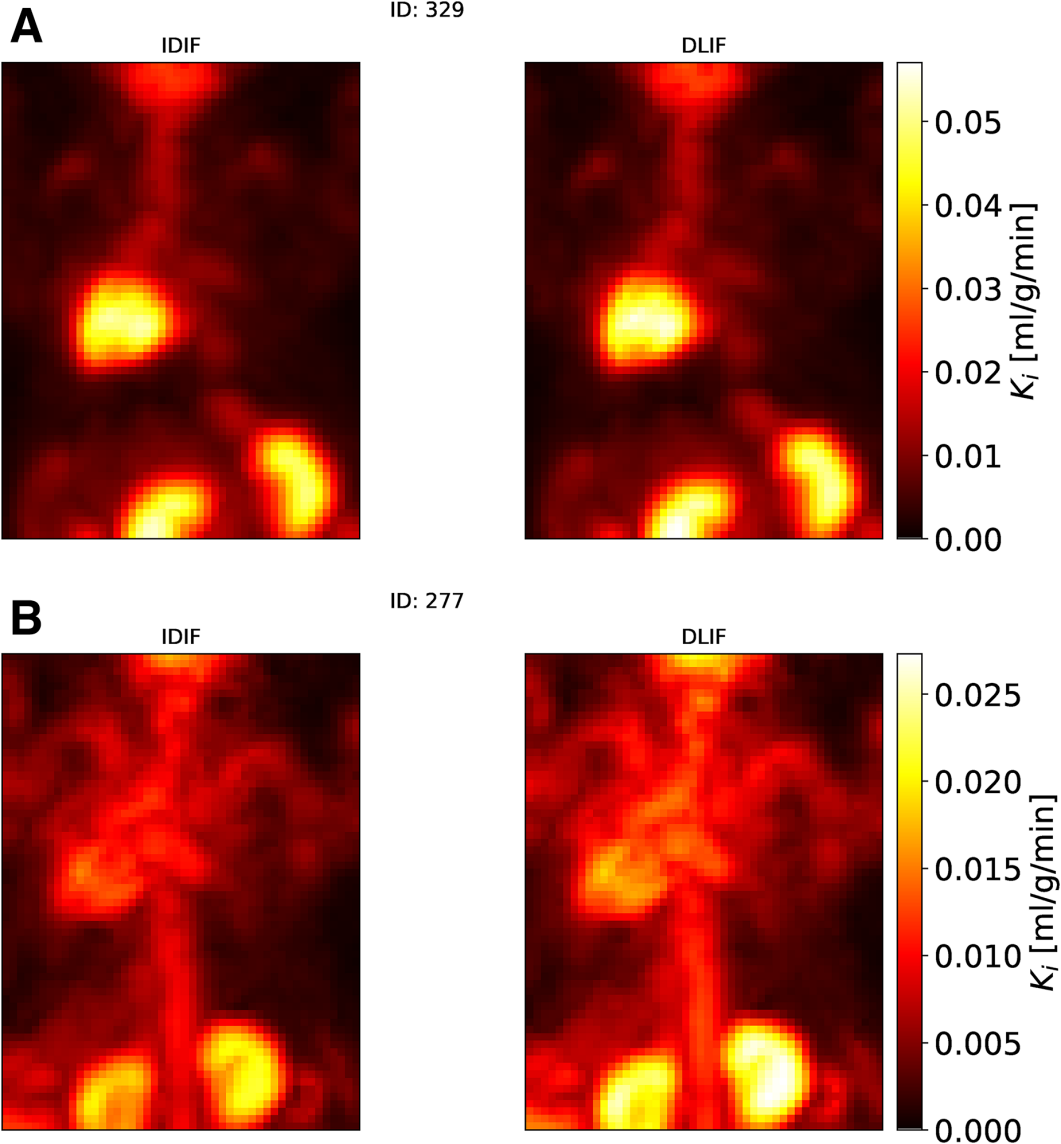
Maximum intensity projections of voxel-wise Patlak images of two mouse scans from the UiT dataset using IDIF and DLIF as input function, respectively. (**A**) The mouse scan with the minimum error (Figure 2A). (**B**) The mouse scan with the maximum error (Figure 2C).

Following DLIF prediction, Patlak modeling was performed to investigate the potential usefulness of the DLIF model for glucose metabolic rate measurements. Figure 3B displays all voxel-wise $K_{i}$ data points as scatterplot, using DLIF and IDIF as input function, respectively, while Table 2 and Supplementary Figure S2 presents statistics and data from the regional $K_{i}$ values, for brain and myocardium tissues, respectively. The voxel-wise influx rate constants displayed a strong linear relationship (slope: 1.05) and a strong correlation (correlation coefficient: 0.94) for the majority of the mouse scans. A few outlier cases are obvious as colored data points deviating from the identity line in Figure 3B. Nevertheless, visual comparison of the voxel-wise influx rate constants calculated with IDIF and DLIF, respectively, for the mouse scan with minimum and maximum errors from Figure 2, still show promising similarities (Figure 4). The regional Patlak analysis (Table 2 and Supplementary Figure S2) indicated good agreement between the population average influx rate constants for brain and myocardium tissues (average errors: ≤10%) and a strong correlation (correlation coefficient: 0.78–0.95). The obtained P values, surpassing the significance level, suggested insufficient evidence to reject the null hypothesis of significant differences between the groups.

**Table 2:** Comparison of $K_{i}$ calculated from the reference input function and from the DLIF model for the UiT and UdS datasets. For the UdS dataset, the comparison of $K_{i}$ from the reference AIF and from the IDIF is also shown. Further details from these data are shown in Supplementary Figures S2 through S4. Note that the identified outliers, indicated in Table S1 and S2, were excluded in the calculation of the table.

Dataset	Model	Statistics of $K_{i}^{*}$	Brain	Myocardium
UiT	Reference IDIF	Estimate (ml/g/min)	0.0176±0.0019	0.123±0.020
	DLIF	Estimate (ml/g/min)	0.0187±0.0024	0.129±0.021
		Average error (%)	10±8	5±5
		Correlation coefficient	0.78	0.95
		t test P value	0.15	0.09
UdS	Reference AIF	Estimate (ml/g/min)	0.0155±0.0075	0.141±0.063
	DLIF	Estimate (ml/g/min)	0.0152±0.0043	0.125±0.039
		Average error (%)	20±39	−3±20
		Correlation coefficient	0.94	0.91
		t test P value	0.87	0.38
	IDIF	Estimate (ml/g/min)	0.0132±0.0055	0.111±0.039
		Average error (%)	−9±22	−17±10
		Correlation coefficient	0.84	0.97
		t test P value	0.36	0.09

^∗^Estimate (ml/g/min) and average error (%) are expressed as mean ±1 confidence interval. Average error was calulated as $\frac{1}{N}\sum_{i=1}^{N}(\frac{K_{i,\mathrm{AIF}}-K_{i,\mathrm{DLIF}}}{K_{i,\mathrm{AIF}}})\times 100$. Correlation coefficient, and P values are calculated from ($K_{i,\mathrm{DLIF}}$, $K_{i,\mathrm{Reference}}$) pairs within each dataset.

### 3.2 External evaluation

**Figure 5:**
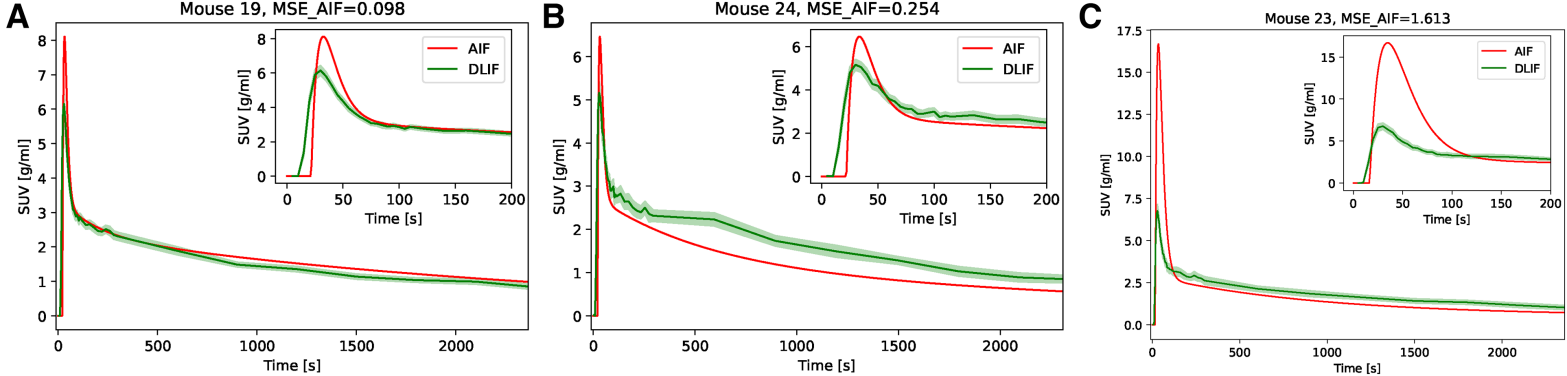
AIF and predicted DLIF for the external test data set. (**A**) AIF and mean ±1 confidence interval DLIF for the mouse scan with the lowest error. (**B**) AIF and mean ±1 confidence interval DLIF for the mouse scan with the 50-percentile error. In (**A**) and (**B**), the mean DLIF was calculated over the training runs (n=50). (**C**) AIF and mean ±1 confidence interval DLIF for the mouse scan with the largest error.

**Figure 6:**
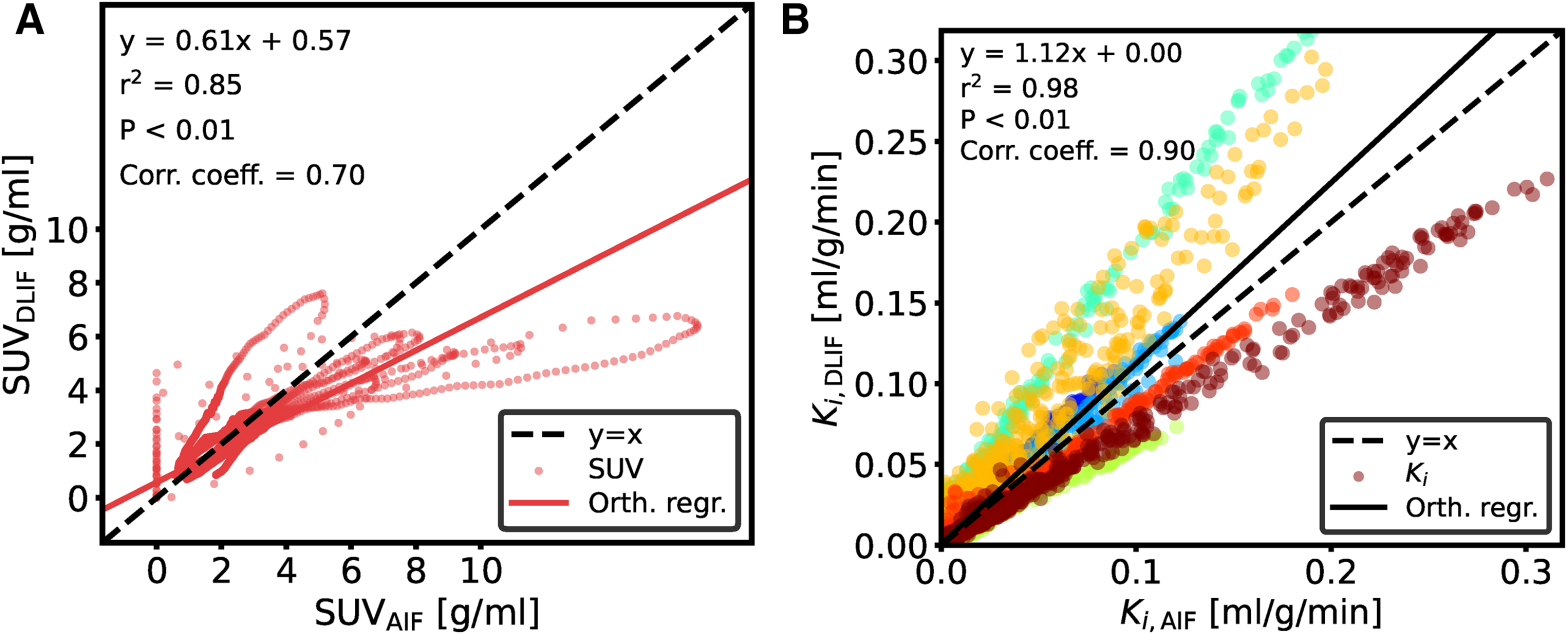
Comparison of data points for UdS data between (**A**) DLIF and AIF curves and (**B**) voxelwise Patlak using DLIF and AIF as input function, respectively. Model training was repeated 50 times and the data points shown are the average over the runs. DLIF was predicted for each mouse scan when it was in the test set during each fold. Due to large number of data points in (**B**), for visualization purposes, only 10,000 randomly sampled data points are shown. The color cycle indicates each mouse scan.

A DLIF model trained on the full UiT dataset was applied to the UdS data for the purpose of external evaluation. The general curve shape is captured by the DLIF model and is in good agreement for some mouse scans (Figure 5A). However, there are also examples where the DLIF model fails to predict the tail (Figure 5B) or the peak (Figure 5C) in this external dataset. The predictions for all test mouse scans are shown in Supplementary Figure S1. For most mouse scans, the DLIF model is unable to predict the AIF peak, while the tail regions are generally in better agreement with the mean DLIF curve (Supplementary Figure S1). This is also visible in the comparison of individual data points (Figure 6A). There is a strong correlation between the data points (correlation coefficient: 0.70), with a linear tendency (slope: 0.61), although with discrepancies, especially around the peak, corresponding to high data point values.

**Figure 7:**
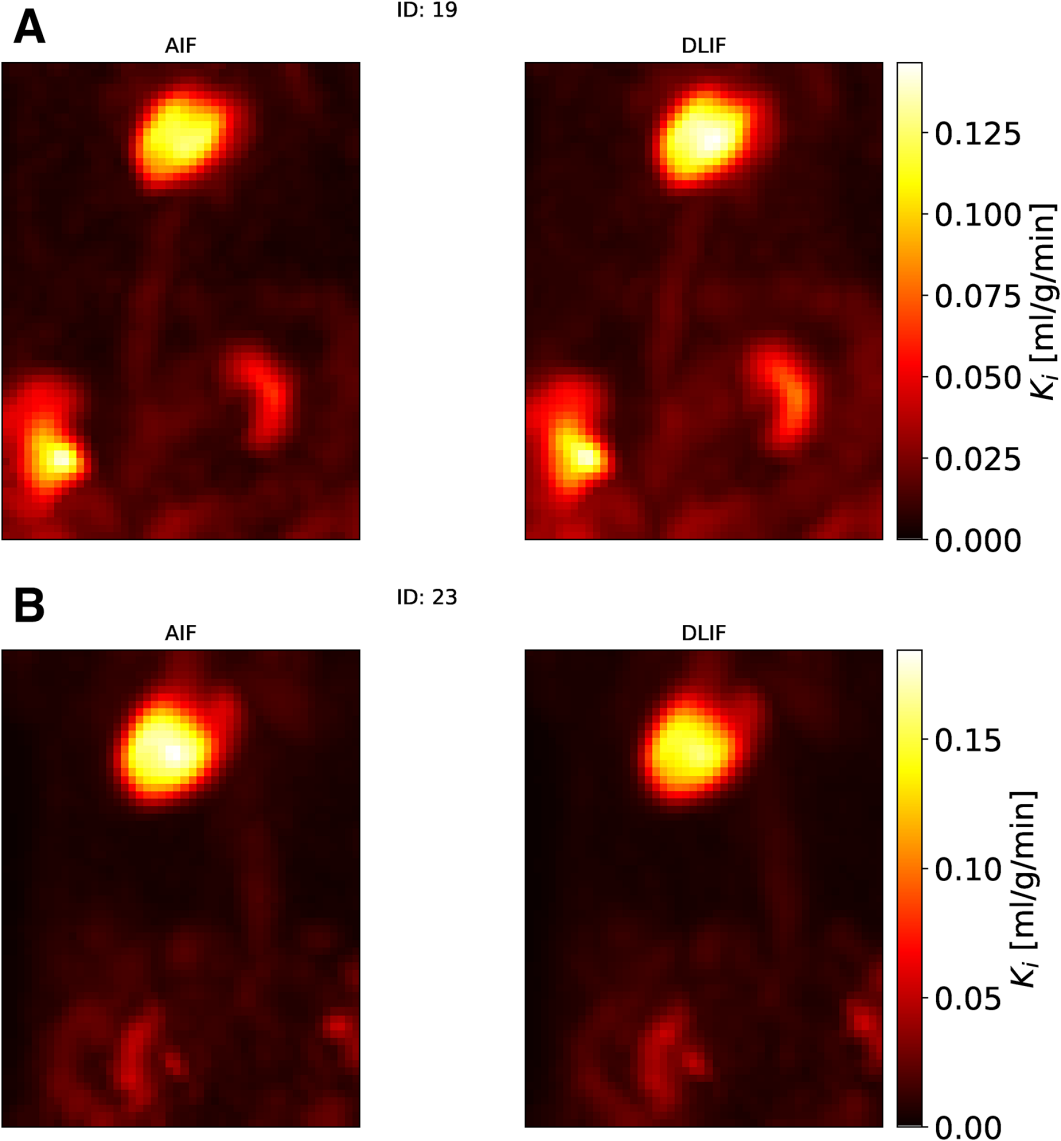
Maximum intensity projections of voxel-wise Patlak images of two mouse scans from the UdS dataset using AIF and DLIF as input function, respectively. (**A**) The mouse scan with the minimum error (Figure 5A). (**B**) The mouse scan with the maximum error (Figure 5C).

The influx rate constant from Patlak modeling, obtained using DLIF and reference AIF as input function, respectively, showed a strong linear relationship (slope: 1.12) and a strong correlation (correlation coefficient: 0.90) for the voxel-wise calculations (Figure 6B). Visual comparison of the voxel-wise influx rate constants calculated with AIF and DLIF, respectively, for the mouse scan with minimum and maximum errors from Figure 5, also show promising similarities (Figure 7). The regional comparisons for brain and myocardium tissues (Table 2 and Supplementary Figure S3) indicated average errors of 20% and−3%, respectively, with a strong correlation (correlation coefficient: 0.91–0.94). The obtained P values for brain and myocardium tissues, surpassing the significance level, suggested insufficient evidence to reject the null hypothesis of significant differences between the groups. Table 2 and Supplementary Figure S4 also displays the corresponding comparisons between $K_{i}$ obtained with the reference AIF and the one obtained using the IDIF in the UdS dataset. These results indicate similar agreement for the brain region compared to DLIF (mean error: −9%, correlation coefficient: 0.84, P value: 0.36), while for myocardium tissues, larger and significant deviations from the reference AIF were obtained, compared to DLIF (mean error: −17%, correlation coefficient: 0.97, P value: 0.09).

## 4 Discussion

Tracer kinetic modeling from dynamic PET imaging requires accurate knowledge of the AIF, ideally determined through arterial blood sampling. The aim of the current study was to develop and evaluate a deep-learning-based prediction model, DLIF, that takes directly the four-dimensional PET data as input, in order to predict a usable input function.

DLIF offers several other advantages relative to currently available methods for AIF estimation. Compared to arterial blood sampling, a trained DLIF model is a non-invasive method, implying simple and convenient use, without the need for surgery, thus allowing longitudinal PET experiments in mice. Compared to our previously published machine learning derived input function (MLDIF) (25, 26), DLIF reduces the subjective bias and preprocessing time by avoiding the need for manual extraction of input time-activity curves. Other common AIF estimation methods, including IDIF (10, 19–21) or simultaneous estimation (17, 18, 22, 23), require correction for the partial volume effect, which is non-trivial, scanner-dependent, and must be carefully measured, or still require a late blood sample for calibration purposes. In contrast, our experiments indicate that a trained DLIF model allows the prediction of both the shape and the amplitude of an input function, using solely image-derived input data, thus without the need for a blood sample for AIF scaling.

### 4.1 Cross-validation

We first trained and evaluated the DLIF model on 68 mouse scans with IDIF targets using cross-validation. These experiments indicated that DLIF could capture the overall input function curve shape, with an early peak and a vanishing tail, and as such, could be a useful approach for estimating an IDIF (Figure 2). Data points between DLIF and IDIF input functions showed a linear behavior and were highly correlated (Figure 3A). As the input function curve itself is not the interesting result in most dynamic PET studies, we evaluated the influx rate constant, $K_{i}$, from Patlak modeling using the reference IDIF as input function, and compared it to the corresponding $K_{i}$, when using DLIF as input function. This analysis was done on both voxel-wise (Figures 3B and 4), and on a regional level for brain and myocardium tissues (Table 2, Supplementary Figure S2). In the voxel-wise scatterplot (Figure 3B), each mouse scan is displayed with a different color. The individual slope coefficients for the majority of mouse scans were around 1, however, 10 (15%) of the 68 mouse scans had more severe deviations from the identity line, with slope values outside the range of 1±0.4 (Supplementary Table S1). One of these was attributed to tracer injection problems (yellow data points in Figure 3B). Six of the 10 outliers (60%) were BALB/c mice. This strain was represented by only 17 (25%) of the 68 mouse scans in the training data. It is well known that tracer kinetics and glucose metabolism can vary significantly between different mouse strains, especially for diseased strains (40, 41). Based on the unbalanced number of samples from the two strains, we hypothesize that the DLIF model is sensitive to differences in uptake pattern between different mouse strains, and thus is unable to learn the possible variations among the samples. Among the remaining 3 outliers, 2 mouse scans were attributed to significantly higher myocardium uptake, which could partly explain why the DLIF model was less accurate for these samples. The remaining outlier had an unusual positioning in the mouse bed with its spine being curled, and not elongated, as for most other mice, which could affect the DLIF model output (42). Also note that the small P-values found in Figure 3 (P<0.01) are expected for comparisons of large sample sizes (43).

The regional analysis indicated similar mean values for brain and myocardium tissues, with mean errors below 10 %, strong correlation (correlation coefficients: brain: 0.78, myocardium: 0.95), and non-significant differences between the influx rate constants derived using DLIF and reference IDIF as input function (Table 2 and Supplementary Figure S3). These findings were similar to our previously published MLDIF model for brain, where we reported correlation coefficients of 0.56 for brain, and 0.90 for myocardium, with average errors of 7% and 4%, respectively [Table 3 in (25)]. The MLDIF model, however, was based on extracted time-activity curves from 5 manually defined tissue regions (myocardium, brain, liver, muscle and brown fat) as model input. Although the results were similar when using DLIF instead of MLDIF, DLIF undoubtedly overcomes the time-consuming need for manual tissue region delineation in the input images. This removes potential bias in the delineation step, and significantly simplifies the processing pipeline, as well as shortens the application time required to use the DLIF model.

### 4.2 External evaluation

The DLIF model, was trained with reference IDIF data targets due to the limited availability of high quality and high quantity small animal PET datasets with ground truth arterial blood sampling. To investigate the potential application of DLIF to predict a useful blood input function, we performed model training on the full 68 mouse scan dataset with IDIF targets, and subsequently evaluated the performance on the external UdS dataset, which contained continuous arterial blood samples as ground truth. The predicted DLIF curves were in good agreement with the ground truth AIF for some mouse scans in the UdS dataset (Figure 5), while there were larger discrepancies for others (Supplementary Figure S1). In general, the tail of the DLIF prediction was in better agreement compared to the peak. This discrepancy could be explained by a larger variation in the peak region for the UdS data (Figure 6A). There were several systematic differences in the data collection methodology between the UiT training data and the UdS test data, which could explain these deviations. Most importantly, the UdS mice were not fasted before the experiments, which is reflected in the slightly higher blood glucose values of the UdS data. It is well known that fasting affects the glucose metabolism in mice, and contributes to reducing the variability between samples (44–47), and specifically, myocardium uptake is affected by fasting, a region that we have shown is important for the prediction of the input function (25). Another difference between the studies was that the injection volume in the UdS data was not fixed. Four mice were injected using 100 μl, while four mice were injected with 200 μl. This volume difference could most likely introduce a variability in the peak of the input function, which was not accounted for in the training data used for the DLIF model. Also, for the UdS data, no attenuation or scatter correction was performed, because an associated CT scan was missing from the dataset. This could introduce underestimations of the measured activity concentration depending on tissue position (48), something which is not accounted for by the DLIF model. Lastly, the UdS test mice were of three different strains, and four of the mice could not be traced to a specific animal supplier, which could introduce additional bias in the data.

Despite these discrepancies, following Patlak modeling, the voxel-wise influx rate constants obtained using DLIF and reference AIF as input function for the UdS data showed an overall strong linear relationship and strong correlation (Figure 6B), even though systematic deviations are obvious for different mice. We found small but systematic underestimations of the slope coefficient for all BALB/c mice (n=3, average slope coefficient: 0.8), while the slope coefficients for C57/BL/6 (n=2, average slope coefficient: 1.4) and CD-1 (n=3, average slope coefficient: 1.5) were larger and systematically overestimated (Supplementary Table S2). While 17 (25%) of the 68 mouse scans used for training of the DLIF model were from the BALB/c strain, the C57/BL/6 and CD-1 strains were not represented among the training data. Similar to our discussion around Figure 3B, this further indicates that the DLIF model is sensitive to uptake patterns in mouse strains that were not part of the training data.

As evident from Figure 6A, the lower SUV values originating from the input function tail are closer to the identity line, compared to larger SUV values, belonging to the peak. The linear fit during Patlak graphical analysis is based on the steady state part following the input function peak (6). Although the integral of the full input function is present in the equation, the impact of the peak on the $K_{i}$ is minimal, and consequently, this method is robust to noise and bias around the input function peak. This could explain why the Patlak $K_{i}$ scatterplot (Figure 6B) still showed high correlation in the voxelwise analysis, despite the discrepancy of many of the peak regions (Supplementary Figure S1). The regional comparison of $K_{i}$ calculated using reference AIF and DLIF (Table 2 and Supplementary Figure S2) indicated mean errors of 20% and −3% in $K_{i}$ for brain and myocardium regions, respectively, with a strong correlation, and non-significant differences between the groups. These differences could be expected because of the mentioned differences between the UiT training data, and the UdS test data. Still, while DLIF-based calculation of $K_{i}$ showed slightly larger errors for brain tissue, compared to IDIF, both results were non-significantly different from the influx derived with the AIF. For myocardium tissue, the average error in the DLIF-based calculation of $K_{i}$ was similar to the error obtained during cross-validation experiments, while the corresponding error for the IDIF-based calculation was larger and significantly different from the AIF-based influx. Interestingly, although IDIF is a commonly used method for AIF estimation in the literature (16, 49), our results indicate that there might still be significant discrepancies between IDIF and AIF for myocardium tissues, which could be overcome by using the DLIF method. Again, we note that the small P-values found in Figure 6 (P<0.01) are expected for comparisons of large sample sizes (43). With all these mentioned limitations and differences between the UiT training data and the UdS test data, we argue that the DLIF model trained on IDIF reference targets still shows promising potential when compared to the external UdS data with reference AIF targets.

### 4.3 Limitations

As depicted in Figures 3A and 6A, the DLIF model sometimes over- or underestimates the reference input function in early time frames, evident as vertical and horizontal data points around t=0, respectively. For these cases, we hypothesize that the DLIF model is unable to handle slight time-shifts in the input data. This will be investigated in future research. Our results furthermore indicate that the DLIF approach is sensitive to the specific mouse strain that it was trained on. Further prerequisites for the DLIF approach is that representative training data have been collected for the specific tracer, imaging system, and imaging protocol.

### 4.4 Future work

Although our work provided a comparison between DLIF and an AIF in the UdS test data, these findings must be further investigated in future research because of the large differences between the experimental methods in the UiT and UdS datasets. For instance, future work could include studying the dependency of the DLIF model to factors such as tracer, mouse strain, and variations in different experimental conditions and imaging protocols. Future research must also evaluate a DLIF model trained on a reference AIF measured in arterial blood.

The DLIF approach was in this work evaluated with [^18^F]FDG on a specific small animal PET imaging system. With further comprehensive validation, we suggest that the DLIF model could be retrained for other tracers and imaging systems. It is also conceivable that tracers requiring metabolite-correction may be modelled. DLIF could also have relevant applications in clinical human PET imaging (26). The accuracy of the DLIF models for a particular PET application will, in the end, depend on the quality, quantity and relevance of the available training data. Nevertheless, if properly validated, DLIF could provide a simplified, low-bias method for performing quantitative and longitudinal PET imaging studies in mice.

### 4.5 Conclusion

In conclusion, we demonstrated that our non-invasive DLIF prediction method may be a viable alternative to arterial blood sampling in [^18^F]FDG imaging of mice. The proposed approach does not require manual segmentation for model input, and it is not depending on a late calibration blood sample or any partial-volume correction. The resulting influx rate constants from Patlak modeling agreed well with image-derived reference values and promising agreement was obtained when comparing to data with continuous arterial blood sampling. With further validation, DLIF could overcome the need for arterial cannulation and allow fully quantitative and longitudinal experiments in PET imaging studies of mice.

## Data Availability

The data analyzed in this study is subject to the following licenses/restrictions: Protection of intellectual property and innovation. Requests to access these datasets should be directed to samuel.kuttner@uit.no.
