# Supplementary material for: Deep-learning-derived input function in dynamic [18F]FDG PET imaging of mice
Source: Front Nucl Med. 2024 Apr 11;4:1372379. doi: 10.3389/fnume.2024.1372379 (PMC11460089; doi:10.3389/fnume.2024.1372379)
Supplement: Supplementary file 1 [file Datasheet1.pdf]

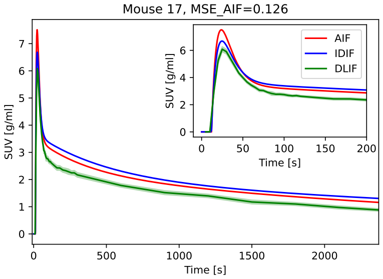

(a)

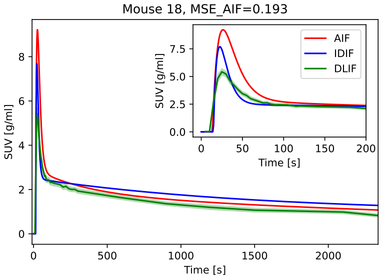

(b)

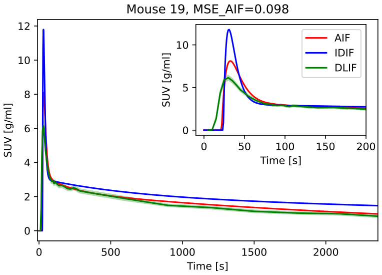

(c)

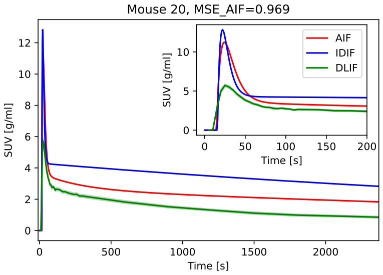

(d)

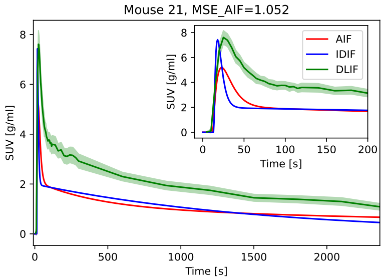

(e)

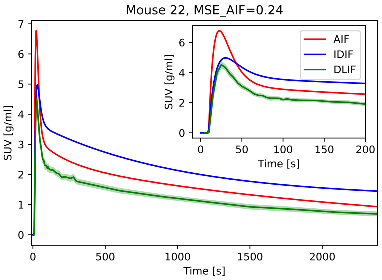

(f)

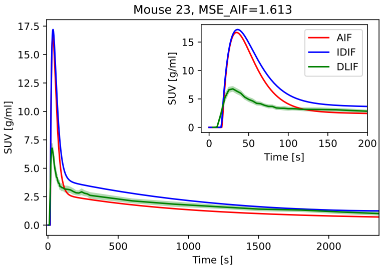

(g)

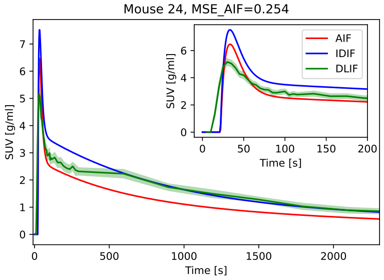

(h)

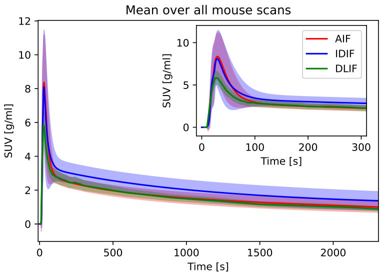

(i)

Figure S1: (a) through (h): AIF, IDIF, and DLIF for the 8 test mouse scans in the UdS dataset. The DLIF data are shown as mean ± 1 confidence interval over all trained models ($n = 50$). (i): Mean ± 1 confidence interval AIF and DLIF over all mouse scans ($n = 8$). Subfigures (c), (h) and (i) in this figure are identical to Figure 4a, b and c in the main paper, respectively, but here also showing the IDIF data.

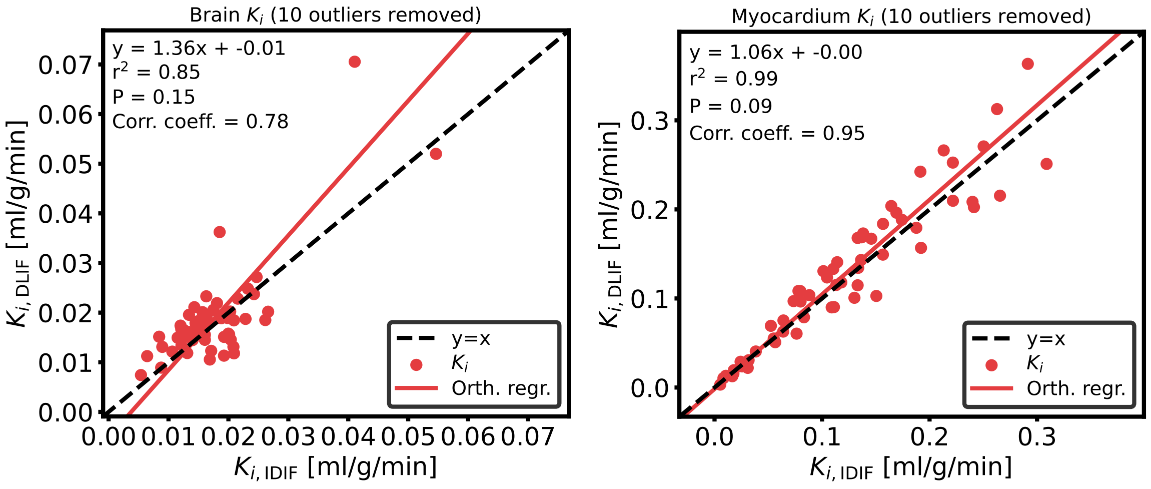

(a) (b)

Figure S2: Comparison of influx rate constants, K_i, from regional Patlak analysis using DLIF and IDIF as input function in the UiT dataset. (a) K_i for brain tissue. (b) K_i for myocardium tissue. Outliers, indicated with an asterisk in Table S1, have been excluded in this figure.

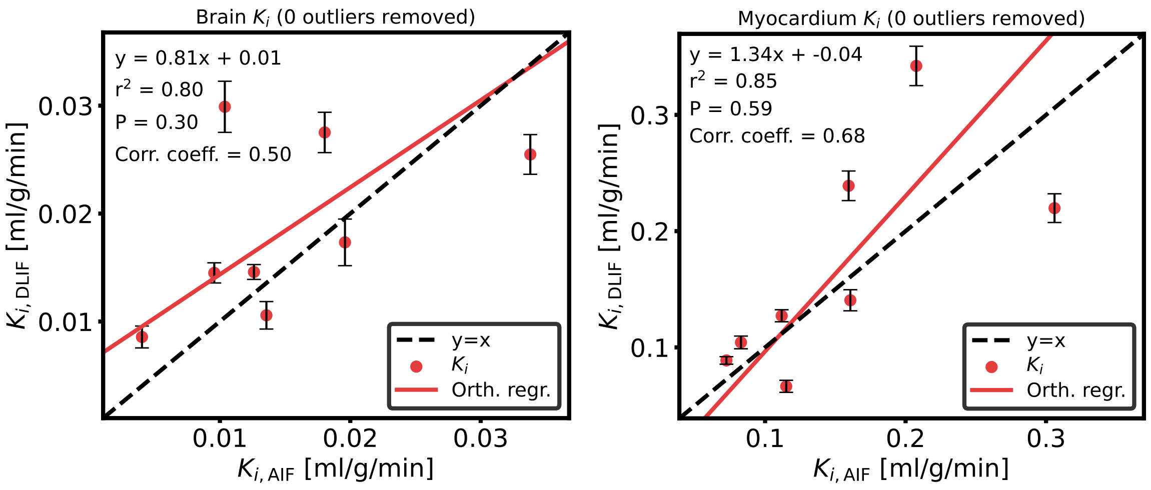

(a) (b)

Figure S3: Comparison of influx rate constants, K_i, from regional Patlak analysis using DLIF and AIF as input function in the UdS dataset. (a) K_i for brain tissue. (b) K_i for myocardium tissue. The mean DLIF was calculated over the training runs ($n = 50$). Error bars indicate mean $\pm$ 1 confidence interval for each mouse scan. Outliers, indicated with an asterisk in Table S2, have been excluded in this figure.

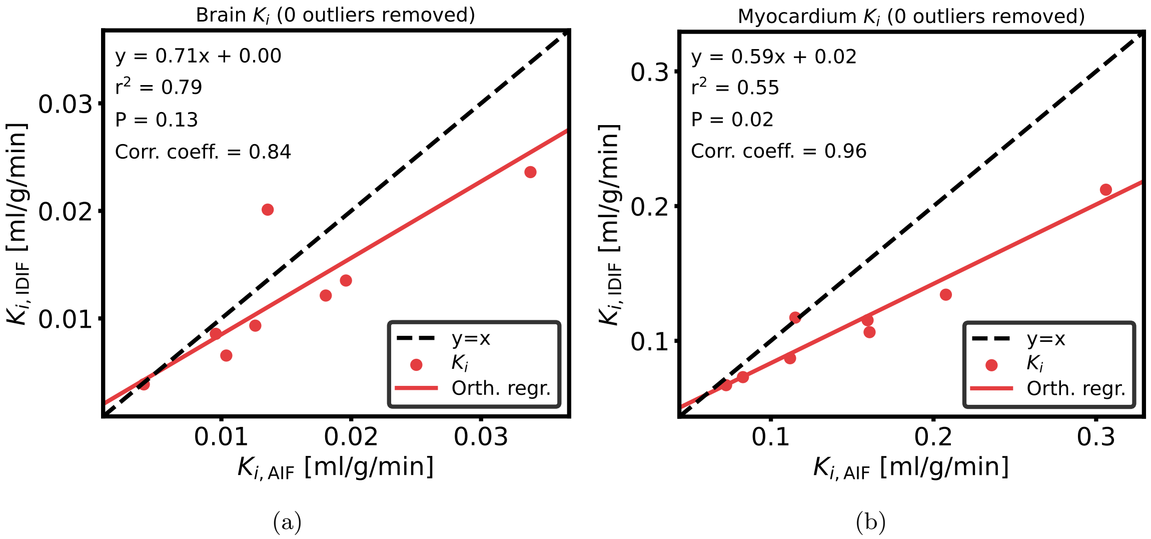

Figure S4: Comparison of influx rate constants, K_i, from regional Patlak analysis using IDIF and AIF as input function in the UdS dataset. (a) K_i for brain tissue. (b) K_i for myocardium tissue. Outliers, indicated with an asterisk in Table S2, have been excluded in this figure.

Table S1: Slope coefficient, r^2, P value and correlation coefficient for each mouse scan of the UiT dataset from the regression analysis shown in Figure 3b in the main paper. Slope coefficients outside the range of 1 ± 0.4 are indicated with and asterisk (*).

Mouse scan	Slope	r^2	P	Correlation coefficient	Mouse strain
224	0.7	0.869	0.0	0.998	NZBWF1
225	0.9	0.992	0.0	0.998	NZBWF1
227	1.4	0.73	0.0	0.996	NZBWF1
229	1.2	0.954	0.0	0.997	NZBWF1
230	0.9	0.989	0.0	0.992	NZBWF1
232	2.6*	-2.923	0.0	0.806	BALB/c
233	0.7	0.869	0.0	0.977	NZBWF1
235	0.7	0.873	0.0	0.987	BALB/c
237	0.9	0.994	0.0	0.998	NZBWF1
238	1.3	0.905	0.0	0.996	NZBWF1
239	1.2	0.803	0.0	0.97	NZBWF1
243	1.5*	0.61	0.0	0.981	BALB/c
249	1.0	0.998	0.0	0.998	NZBWF1
251	1.4	0.766	0.0	0.96	NZBWF1
264	1.0	0.998	0.0	0.999	NZBWF1
265	1.6*	0.367	0.0	0.974	NZBWF1
268	0.8	0.938	0.0	0.998	NZBWF1
269	0.6	0.789	0.0	0.998	BALB/c
270	0.8	0.964	0.0	0.999	NZBWF1
272	0.8	0.918	0.0	0.998	NZBWF1
274	0.8	0.941	0.0	0.998	NZBWF1
276	0.8	0.96	0.0	0.998	NZBWF1
277	1.3	0.871	0.0	0.998	BALB/c
278	1.2	0.927	0.0	0.996	BALB/c
281	0.8	0.954	0.0	0.999	NZBWF1
282	1.2	0.971	0.0	0.999	NZBWF1
283	1.3	0.888	0.0	0.996	NZBWF1
286	1.1	0.967	0.0	0.999	NZBWF1
288	0.9	0.991	0.0	0.993	NZBWF1
289	1.5*	0.612	0.0	0.988	NZBWF1
290	1.1	0.992	0.0	0.999	NZBWF1
291	1.1	0.984	0.0	0.998	NZBWF1
303	1.2	0.927	0.0	0.999	NZBWF1
304	0.7	0.846	0.0	0.998	NZBWF1
305	1.3	0.882	0.0	0.997	NZBWF1
306	1.2	0.929	0.0	0.994	NZBWF1
307	1.2	0.914	0.0	0.981	NZBWF1
308	1.3	0.756	0.0	0.973	NZBWF1
312	1.4	0.816	0.0	0.992	BALB/c
313	1.6*	0.364	0.0	0.962	BALB/c
314	1.6*	0.417	0.0	0.977	BALB/c
315	1.0	0.935	0.0	0.958	NZBWF1
316	1.2	0.945	0.0	0.999	NZBWF1
317	1.0	0.987	0.0	0.996	NZBWF1
322	0.4*	0.557	0.0	0.998	NZBWF1
323	0.9	0.99	0.0	0.997	BALB/c
324	1.2	0.944	0.0	0.998	NZBWF1
328	1.2	0.962	0.0	1.0	NZBWF1
329	1.0	0.999	0.0	0.999	BALB/c
332	1.2	0.927	0.0	0.985	BALB/c
333	2.3*	-1.527	0.0	0.929	NZBWF1
334	1.0	0.997	0.0	0.999	NZBWF1
337	1.2	0.941	0.0	0.991	NZBWF1
338	1.0	0.995	0.0	0.981	BALB/c
339	1.0	1.0	0.0	1.0	NZBWF1
341	1.2	0.975	0.0	0.998	NZBWF1
346	1.3	0.925	0.0	0.999	NZBWF1
347	1.4	0.737	0.0	0.987	NZBWF1
348	2.1*	-1.015	0.0	0.893	BALB/c
353	1.2	0.936	0.0	0.999	NZBWF1
354	1.1	0.983	0.0	0.979	BALB/c
355	0.9	0.992	0.0	0.998	NZBWF1
358	1.1	0.977	0.0	0.999	NZBWF1
359	0.8	0.944	0.0	0.998	NZBWF1
360	4.3*	-16.55	0.0	0.816	BALB/c
363	1.2	0.944	0.0	0.999	NZBWF1
364	1.0	1.0	0.0	1.0	BALB/c
365	1.2	0.928	0.0	0.999	NZBWF1

Table S2: Slope coefficient, r^2, P value and correlation coefficient for each mouse scan of the UdS dataset from the regression analysis shown in Figure 5b in the main paper. Slope coefficients outside the range of 1 ± 0.4 are indicated with and asterisk (*).

Mouse scan	Slope	r^2	P	Correlation coefficient	Mouse strain
17	1.4	0.802	0.0	0.979	C57/BL/6
18	1.4	0.79	0.0	0.974	C57/BL/6
19	1.1	0.96	0.0	0.988	CD-1
20	2.0*	-0.136	0.0	0.963	CD-1
21	0.7	0.87	0.0	0.969	BALB/c
22	1.5*	0.615	0.0	0.975	CD-1
23	0.9	0.982	0.0	0.994	BALB/c
24	0.7	0.928	0.0	0.997	BALB/c
